# Enhanced recovery after surgery: Narrative review in gynecologic pelvic exenterations

**DOI:** 10.3389/fonc.2025.1728831

**Published:** 2025-12-12

**Authors:** Abigail Barger, Sarah P. Huepenbecker

**Affiliations:** Gynecologic Oncology, St. Luke’s University Health Network, Bethlehem, PA, United States

**Keywords:** ERAS, enhanced recovery after surgery, pelvic exenteration, perioperative, postoperative, gynecologic oncology, surgery

## Abstract

In this narrative review, we discuss enhanced recovery after surgery (ERAS) in pelvic exenterations from a gynecology oncology perspective. There is limited evidence regarding perioperative guidelines for this complex surgical procedure. We will review ERAS societal guidelines, collaborative reviews, expert opinions and provide tables summarizing the pre-admission, preoperative, intraoperative, and postoperative guidelines provided by these sources. We will look at how ERAS protocols have been used in published studies. Lastly, we discuss potential future directions for perioperative care in pelvic exenterations.

## Introduction

Pelvic exenteration (PE) is a radical surgical procedure performed with curative intent for persistent or recurrent centrally located cancers of the pelvis including the cervix, vagina/vulva, uterus, bladder and rectum/anus (1). PE includes en bloc resection of affected and surrounding pelvic organs which may include the removal of some or all the uterus, vagina, vulva, bladder, rectum and anus (2, 3) and may include the peritoneum, muscle and vasculature such as the hypogastric arteries (4). PE can also include plastic reconstruction with perineal reconstruction or creation of a neovagina with various techniques. With the goal of complete resection (R0), obtaining negative surgical margins is imperative (4). In colorectal cancer, a similar procedure, total mesorectal excision (TME), entails removal of the rectum and surrounding structures to include lymph, possibly anus, and peritoneum (5–7). It can also include the removal of pelvic organs to achieve negative margins (5). Similarly, in bladder cancers, a radical cystectomy is done to remove the bladder and surrounding tissue, sometimes including the vagina and uterus, to achieve negative margins (8).

In a national study looking at surgical outcomes, there were 2,305 pelvic exenterations performed from 2005 to 2016, with 15% (n=335) done for gynecologic indications. These procedures included urinary diversion in 99% and bowel diversion in 7.8% of the gynecologic PE (9). In a systematic review looking at survival data for total PE, authors reported in gynecologic cancers PE resulted in 5-year survival of 6-64%, and in colorectal cancer PE resulted in 5-year survival of 8-92% (10). Given the survival outcomes, PE is a viable option for the appropriate patient, as there are limited proven alternatives with curative intent in these patients, such as targeted therapies, immunotherapy, and radiation (7, 11). To continue to offer these procedures, limiting risks for post-operative complications is a valuable discussion.

This review will focus on total pelvic exenteration procedures, primarily in gynecologic oncology. This very morbid surgery has reported complication rates between 30-100% (12–17) and mortality rates between 2-3% (9, 12, 18). Often PE is done in the recurrent setting with 57-97% of patients having undergone prior pelvic radiation (3, 13, 14), furthering the surgical complexity of this procedure (3, 19, 20). Given the complexity, high morbidity, and potential mortality, pelvic exenterations require a multidisciplinary team approach which may include gynecologic oncology, colorectal surgery, urology, plastic surgery, vascular surgery, and anesthesiology (2). In a nationwide study of 1,912 exenterations over 10 years at 181 centers, they reported that the majority of PE performing centers had at least 1 PE per year (21). This study suggested that lower volume centers had worse mortality, but nationwide mortality rates have improved over time (21). With the rarity and the need for the collaboration of multiple surgical subspecialties for this morbid procedure, it is imperative to optimize protocols to ensure patient safety and optimize survival.

Enhanced Recovery After Surgery (ERAS) are multimodal perioperative pathways created to decrease operative morbidity and mortality (22, 23). ERAS protocols have become the standard of care across many surgical specialties, including gynecologic oncology (24–27). In gynecologic oncology, ERAS protocols recommend multimodal analgesia, goal-directed fluid therapy, early ambulation and feeding, limited abdominal drain use, early Foley catheter removal, and deep vein thrombosis (DVT) prophylaxis (27–32). They have been associated with improved perioperative outcomes including decreased length of stay (LOS), readmission, postoperative complications, and costs (26, 33, 34). However, there is extremely limited data regarding ERAS protocols specific for patients undergoing pelvic exenterations. Thus, this review aims to discuss ERAS pathways in the setting of pelvic exenterations. A summary of the discussed articles can be reviewed in **Tables 1**–**4**.

## ERAS society recommendations

Enhanced Recovery After Surgery (ERAS) has become the standard of care within gynecology, gynecologic oncology, colorectal surgery, and urology, among others (27, 35–37). Given the rarity, complexity, and variability of pelvic exenteration, standardized protocols for ERAS are limited. We will review recommendations from these surgical subspecialties as they specifically relate to PE, noting that there generally remains a gap in ERAS society guidelines for PE.

The ERAS Society publishes guidelines by specialty which are widely adopted globally (38). In 2016, the first gynecologic oncology ERAS pathways were discussed (28, 29). The first discussion of ERAS in pelvic exenteration was in the 2019 ERAS update (30). The authors discussed the importance of pre-admission patient counseling, addressing nutritional status, optimizing glucose control, carbohydrate loading, and preventing surgical site infection. Once intraoperative, advanced hemodynamic monitoring with an arterial line, and non-specific fluid management are recommended. Post-operatively, early nutrition, thromboembolic prophylaxis, and early mobilization are supported. The authors also agree with the need for more research using ERAS protocols in PE (30). In the 2023 update, there were no additional ERAS recommendations for PE (31).

Colorectal surgery has been a leader in the ERAS initiative (39, 40). Unfortunately, through the last 25 years of ERAS pathways, there have been no official recommendations from colorectal ERAS Society for the pelvic exenteration, including in most recent update at the time of this article in 2025 (36, 41, 42). They do discuss recommendations for colorectal resections but not specific to PE or mesorectal excision.

Likewise, urology ERAS pathways do not have specific pelvic exenteration recommendations for review at this time (8, 37). There are, however, ERAS recommendations for patients undergoing a radical cystectomy for bladder cancer (8). They generally align with the Gynecologic Oncology ERAS guidelines. In addition to those recommendations, they additionally mention correction of anemia, tobacco and alcohol cessation for at least 4 weeks, preoperative exercise routine, and avoidance of bowel preparation preoperatively. Intraoperatively, they support the use of thoracic epidurals for pain control, extrapolating outcomes in urology and colorectal surgery. Fluids are managed with an individualized goal directed method, noting measurement of urine output is often difficult during these procedures. They support the use of an abdominal drain if a cystectomy is performed to assist with identification of urinary leak, although there is no high-quality evidence to support routine use. If a bladder resection is not performed, the foley catheter should be removed by POD1. For prevention of ileus, they support multimodal antiemetics, chewing gum, and magnesium. The duration for removal of ureteral stents and transurethral catheter for a neo-bladder is not established (8). While not directly applicable to gynecologic PE, ERAS protocols for radical cystectomies provide useful insight and can provide additional suggestions within the limited published societal guidelines.

## Collaborative group reviews

There are two published reviews of pelvic exenteration guidelines; one from the PelvEx Collaborative and the other from an Australian anesthesiology group (2, 43). The PelvEx Collaborative is an international group comprised of members across five continents devoted to research and outcomes of patients who undergo pelvic exenteration. They published recommended guidelines in 2022 (2). It outlines the patient selection, procedure details, staging, preoperative optimization, perioperative recommendations, surgical approaches, postoperative care, follow-up and surveillance for PE. When comparing to other sources (**Tables 1**-**4**), they have several specific features. This includes pre-admission cardiac stress testing if the patient has additional risk factors and preparation of 2 units of red blood cells. They do not make any formal recommendations on preoperative bowel preparation, chemical venous thromboembolism (VTE) prophylaxis, antibiotics, or pain medications. Two dedicated anesthesia colleagues (if >12hrs of surgery anticipated) and goal directed fluid therapy are recommended. Safe patient positioning with lowering of the legs every 2–4 hours is expected. If the bladder was not resected, the foley should be removed by POD2. If a pelvic drain was placed, it should be removed by POD8. In addition to hospital-controlled bundles, the authors stress the importance of social support as part of a patient’s follow-up care. PelvEx continues to be a valuable resource for specialists involved in PE.

**Table 1:** Pre-admission recommendations.

Pre-admission	ERAS Society		Collaborations		Expert Opinions		Published Studies			
Recommendation	Gyn Onc (30)	Urology – radical cystectomy (8)	PelvEx Guidelines (2)	Anesthesia Guidelines (43)	PelvEx Delphi (44)	TME protocol (45)	Harji et al (13)	Wan et al (47)	Huepenbecker et al. (12)	Nordkamp et al. (46)
Anesthesia Consult			X	X	X					
Cardiopulmonary testing			X	X	X					
Preoperative Labs		X, Screen/correct anemia	X, CBC, BMP, T&C, as needed A1C, LFT	X, Screen/correct anemia		X, Screen/correct anemia	X, Screen/correct anemia (Hgb goal 10g/dL)			X, Screen/correct anemia
Prehabilitation Evaluation		X, Exercise encouraged	X			X				X
Nutrition Assessment	X	X	X			X	X, Supplements 7 days preop			X
Patient education/counseling	X	X				X			X	X
Tobacco and Alcohol Cessation		X, 4 weeks preop				X				X
Glycemic Control	X									

ERAS indicates Enhanced Recovery After Surgery; Gyn Onc, gynecology oncology; CBC, complete blood count; BMP, basic metabolic panel; T&C, type and cross; A1C, hemoglobin A1c; LFT, liver function test; TME, total mesorectal excision.

In a review article from Australia in 2024, they published recommended guidelines from the anesthesiology perspective for patients undergoing PE (43). Their guidelines are similar to the PelvEx group, including availability of blood products and encouraged use of neuraxial or local nerve blocks (2, 43). They stress the importance of patient positioning and mindfulness of changes to position throughout this lengthy procedure. While there are no ERAS society guidelines for anesthesia in PE, this document serves as a starting point to guide management during these surgeries.

## Expert opinion

Due to a lack of high-level evidence evaluating ERAS protocols in pelvic exenterations, most guidance is based on published expert opinion. In 2021, the PelvEx Collaborative published a list of consensus statements of perioperative recommendations using the Delphi method, which is a method of interviewing via questionnaires in several rounds to come to a consensus (44). There was >80% consensus on 34 published statements by the surveyed group which included pelvic exenteration surgeons, radiologists, specialty nurses, medical oncologists, and anesthesiologists. These statements were incorporated into the aforementioned PelvEx published guidelines (2).

A few statements from this global consensus project that did not make it into the PelvEx guidelines include a multidisciplinary team briefing before induction of anesthesia, consideration of using thromboelastography to assist in the management of hemorrhage and planned postoperative ICU admission. The collaborators did not reach a consensus on 14 other statements (44). There was not agreement on admission the night before surgery, central line placement, total IV anesthesia, electroencephalographic/bispectral index monitoring, non-invasive cardiac output monitoring, anticoagulation, massive transfusion protocol, management of SIRS, and the use of prolonged epidural. This document represents expert opinion globally, and serves as a valuable reference given the basis in the PelvEx data and the lack of otherwise defined protocol recommendations.

In Nordkamp et al. in 2024 from the Netherlands, they discuss perioperative protocols for patients undergoing beyond total mesorectal excision (TME) for rectal cancer (45). This procedure involves pelvic exenterations at their institution, comprising 15% of their TMEs. This was a four-phase study consisting of a literature review, retrospective cohort study of ERAS elements, retrospective review of ERAS compliance, and lastly, multidisciplinary creation of an ERAS protocol based on their reviews. The team included five physicians (colorectal surgeon, plastics surgeon, urologist, anesthesiologist, and critical care), physiotherapist, dietician, and multidisciplinary nurses (surgical oncology, oncology, stoma care, and critical care).

Their recommendations are summarized in **Tables 1**-**4** and are overall similar to other literature in the pre-admission and preoperative settings, with encouraged preoperative oral carbohydrate loading and bowel preparation (if anticipating a low anterior resection). For specific prevention of post-op nausea and vomiting, Dexamethasone 8mg IV and Granisetron 2mg IV are used. Intraoperatively, temperature control is supported with forced air heating. Abdominal drains are avoided unless a urinary resection occurs. They provide specific fluid management protocols, use only IV anesthesia, and do not use epidurals. Post-operatively, they have very specific protocols for pain control, VTE prophylaxis, nausea, and ileus prevention (**Table 4**). They also provide a day-by-day protocol for nutrition and mobilization with goals for each day (45). These two articles show a wide variety of recommendations for patients undergoing PE among experts across the world, with multiple protocolized guidelines that can serve as helpful templates for these complex procedures.

## Published studies

There are extremely limited studies evaluating ERAS protocols for patients with pelvic exenterative procedures. There are many studies assessing the validity of ERAS in gynecologic oncology, but most of the studies either excluded patients undergoing pelvic exenteration or did not discuss them (25–27). We will review the limited evidence regarding ERAS protocols in PE patients.

In 2021, Harji et al. performed a prospective cohort study to evaluate the feasibility of implementing an ERAS protocol for PE patients in France (13). They enrolled all patients (n=145) receiving a PE from 2016 to 2020 at their institution. Their ERAS protocol was created with recommendations from the society guidelines for colorectal cancer surgery (36) and their institution’s multidisciplinary PE specialists. They created 10 components that were not previously part of standard of care of all PE patients. Compliance to the 10 components were recorded for each patient. Their measured protocol components include preoperative correction of hemoglobin with goal of >10g/dL achieved by transfusion if necessary, and nutritional optimization encouraging the use of a 7-day nutrition supplement, Oral Impact. Their intraoperative anesthetic protocol was standardized but not discussed in the manuscript. Postoperatively, patients were admitted to the critical care unit, with the goal of being discharged from the unit by POD2. They measured de-escalation of opioids of patient-controlled opioids were discontinued by POD2, and epidural was removed by POD4. Nutrition was initiated by POD3. Foley catheter was removed by POD2, or POD7 if a partial cystectomy occurred, or on POD 30 if a ureteric reconstruction occurred. If a pelvic drain was placed, it was removed by POD8. If there were J-J ureteral stents, they were removed after 3 weeks. Chemoprophylaxis of thromboembolism with LMWH was continued for 30 days. Their mobilization goals included assisted standing and walking by day 1. Sitting goals depended on abdominal flap creation, with goals between POD 3-5 (13).

The primary endpoint was feasibly measured by compliance with the ten ERAS protocol items. They reported median compliance with ERAS components of 70% (13). Their secondary outcomes were patient outcomes including length of stay (LOS), morbidity, complications, mortality, and readmission. Of the 145 patients enrolled, the majority were male (47%), had colorectal disease (80%), open surgery (76%), and prior radiation (56%). Of the 68 women, 75% did not have any perineal reconstruction. They found that higher ERAS compliance (>70%) was associated with shorter LOS (13 *vs* 17 days, p<0.001), less 30-day morbidity (62.5% *vs* 89.0%, p<0.001), and reduced complication severity measured as Clavien-Dindo Grade III-V (16.7% *vs* 39.7%, p<0.001). There was no significant difference in 30-day mortality between the level of ERAS compliance (3 v 0, p =0.08) (13). While conclusions cannot be drawn about effectiveness given the small sample size, this study demonstrates the feasibility of ERAS in PE.

In another feasibility study by Nordkamp et al. in 2023, they used their previously discussed protocol (45) to assess feasibility in their patient undergoing beyond mesorectal excisions, including 32% receiving a total pelvic exenteration (46). After creation and implementation of their perioperative protocol, all patients undergoing TME for rectal cancer in the study period were prospectively included (n=72). The majority were male (67%), all had previous radiation therapy (100%), and most received intraoperative radiation (81%). There was an overall mean compliance of 73.6% with the 39 ERAS components previously described (45, 46). Overall, the median LOS was 9 days and the complication rate was 71%, with 17% being Clavien-Dindo Grade IIIb-V. On average, the abdominal drain was removed by POD3, nasogastric tube removed by POD3, and if possible, the foley catheter was removed by POD6. They did a subgroup analysis comparing those who received multimodal anesthesia with a continuous wound infusion catheter compared to those who received an epidural. The epidural patients used statistically significant more opioids per day on POD1-4 (46). This study again describes the feasibility of using an ERAS protocol in these complex surgical patients, although a large study is required to evaluate clinical effectiveness.

Wan et al. published a retrospective review in 2016 of open gynecologic surgeries (n=454) performed by a single surgeon, evaluating predictors of early discharge in the setting of implementing an ERAS pathway (47). The “high” intervention surgeries (34%, n=152) included radical hysterectomies, pelvic or para-aortic nodal dissection, and exenterations, although the specific number of patients who received a PE was not reported which limits interpretation of their findings. The ERAS components did not include any preadmission recommendations. Preoperatively, they used prophylactic antibiotics (cefazolin 1g) and performed skin and vaginal preparation with iodine. Intraoperatively, they describe mass closure of the abdomen, suture choices, avoidance of abdominal drains, and they prefer to use multimodal pain control with transversus abdominis plane (TAP) block and paracetamol (acetaminophen) with parecoxib. Postoperatively, they use a patient controlled opioid pump with fentanyl through POD1 then switch to oral nonsteroidals with as needed oxycodone. They encourage fluid intake on POD0 and solids by POD1. They encourage gut motility with scheduled polyethelene glycol and senna through discharge. If allowable, the foley catheter is removed by POD1. The patients are encouraged to be out of bed and walk by POD1. They recommend weight based prophylactic enoxaparin while inpatient starting on POD1. Then, depending on their risk factors determines whether the chemical VTE prophylaxis is continued (47).

This retrospective review found that in the setting of the ERAS pathway, overall 74% of patients were able to be discharged early (POD3) (47). High intervention surgery was not a statistically significant risk factor associated with timing of discharge. Of the high intervention group, 33% were able to be discharged by POD3 (47). Patients were more likely to stay more than 3 days if they were older, had a higher ECOG score, had a vertical midline incision, malignancy, longer OR time, or had complications. This study did not report on level of compliance with the ERAS components. Interestingly, this study did not exclude PE patients, but we are unable to draw meaningful conclusions for this population due to the unknown number of included PE patients. This study suggests the ability to apply an ERAS protocol to complex surgical patients, but further evaluation on the PE population is warranted.

In a more recent retrospective cohort study at a single major cancer institution in 2024, Huepenbecker et al. reports perioperative outcomes of PE before and after implementation of an institutional ERAS protocol (12). They identified 74 pelvic exenteration patients pre-ERAS and 31 patients after implementation of ERAS at their institution. Their ERAS protocol used is the same used for all their open gynecologic surgeries (27). The measured ERAS components included were pre-admission preoperative counseling, avoidance of prolonged fasting, and avoidance of mechanical bowel preparation. To avoid prolonged fasting, they encouraged no solids after midnight, and no liquids up to 2 hours before surgery. Pre-operatively, antibiotics, subcutaneous heparin, and medications to prevent post-operative nausea were given. Intraoperatively, they recommend goal-directed fluid therapy, normothermia, and avoidance of routine abdominal drain placement. Post-operatively, they avoid nasogastric tube, prioritize glucose control, avoid fluid overload, and avoid patient-controlled opioid pain-pump or epidural. For multimodal pain control they used acetaminophen, ibuprofen, pregabalin, oxycodone, and as needed hydromorphone IV.

The majority of these patients had prior radiation (66.7%) and underwent a total pelvic exenteration (76.2%) with a vaginal reconstruction (89.5%). They reported a median ERAS compliance of 60% of the 13 measures, with lowest compliance in avoidance of postoperative fluid overload (31%), and multimodal analgesia without PCA or epidural (32%). All other measures were >80% compliant. All patients (100%), pre- or post-ERAS implementation developed at least one post-operative complication within 60 days of surgery. They found a higher complication rate in the ERAS cohort. ERAS cohort patients were more likely to have post-op ileus (38.7% *vs* 10.8%, p=0.002), urinary leak (22.6% *vs* 5.4%, p=0.014), pelvic abscess (35.5% *vs* 10.8%, p=0.005), post-op bleeding (61.3% *vs* 28.4%, p=0.002), and readmission (71.4% *vs* 46.5%, p=0.025). This study suggested that pelvic exenteration remains a highly morbid procedure (12) and that extrapolating modern open gynecologic ERAS protocols to pelvic exenterations may not be suitable for this complex procedure.

## Discussion

After reviewing the literature of perioperative recommendations for patients undergoing pelvic exenterations, there are many components which remain controversial, and our review also identified areas of alignment across available data. The recommendations are summarized in **Tables 1**-**4** (2, 8, 12, 13, 30, 43–47). Pre-admission assessment and patient optimization is widely comprehensive and seems to be agreed upon, detailed in **Table 1**. The majority of sources recommend patient education, nutrition and prehabilitation assessment. Preoperatively, many sources recommend avoiding bowel preparation, support fasting, prophylactic antibiotics, and oral carbohydrate loading, shown in **Table 2**. Intraoperatively, most articles discussed vascular access, temperature control, and avoidance of hypotension. There remains controversy about pain control in these patients. Most of the reviewed articles used neuraxial anesthesia during and after surgery (2, 8, 43, 44). Some avoid epidurals in these cases and prefer only IV anethesia (12, 45, 46). During these very long operations, the most preferred fluid management strategy is goal-directed fluid therapy for volume management (2, 12, 43, 44). Abdominal drains are not routinely recommended (8, 12, 45, 46). Intraoperative recommendations are summarized in **Table 3**. If an abdominal drain is placed, recommendations exist for removal as early as POD2 or later, by POD8 (2, 13, 45). For VTE prophylaxis, LMWH is the anticoagulation of choice most documented (2, 8, 13, 45). There is no consensus on bowel preparation, use of intraoperative nasogastric tube, when to start postoperative solid intake, use of laxatives, and timing of foley catheter removal. Postoperative recommendations are summarized in **Table 4**.

**Table 2:** Pre-operative recommendations.

Pre-operative	ERAS society		Collaborations		Expert opinions		Published studies			
Recommendation	Gyn Onc (30)	Urology – radical cystectomy (8)	PelvEx Guidelines (2)	Anesthesia Guidelines (43)	PelvEx Delphi (44)	TME protocol (45)	Harji et al (13)	Wan et al (47)	Huepenbecker et al. (12)	Nordkamp et al. (46)
Crossmatch for blood			X 2u RBCs, with more available	X	X 2u RBCs, with more available					
Oral carbohydrate loading		X	X			X				X
Bowel Preparation		AVOID				X, If LAR			AVOID	X, If LAR
Fasting		X, 2 hours clears 6 hours solids				X, 2 hours clears 6 hours solids			X, No solids after midnight, clears 2 hours	
SDD						X				X
Abx ppx		X				X		X, cefazolin	X	X
SQH for VTE ppx									X	X
PONV ppx						X, Dexamethasone and granisetron			X	X
Preop pain meds									X, tramadol, pregabalin, celecoxib, acetaminophen	

ERAS indicates Enhanced Recovery After Surgery; Gyn Onc, gynecology oncology; TME, total mesorectal excision; GI, gastrointestinal; SDD, selective digestive decontamination; Abx, antibiotics; ppx, prophylaxis; SQH, subcutaneous heparin; VTE, venous thromboembolism; PONV, postoperative nausea and vomiting; u, units; RBC, red blood cells; LAR, low anterior resection.

**Table 3:** Intraoperative recommendations.

Intraoperative	ERAS Ssciety		Collaborations		Expert opinions		Published studies			
Recommendation	Gyn Onc (30)	Urology – radical cystectomy (8)	PelvEx Guidelines (2)	Anesthesia Guidelines (43)	PelvEx Delphi (44)	TME protocol (45)	Harji et al (13)	Wan et al (47)	Huepenbecker et al. (12)	Nordkamp et al. (46)
Surgical/anesthesia details							X, standardized	X		
Vascular access	X, arterial line		X, large bore PIV, Arterial line	X, central line, arterial line	X, large bore PIV					
Temperature management		X		X, warmed fluids		X, forced air heating			X	X, forced air heating
Lab monitoring			X, blood gases, Hgb, lactate		X, blood gases, Hgb, lactate	X, glucose with insulin correction				
Anesthesia colleagues x2 (>12hr case)			X		X					
Dedicated OR team					X					
Mechanical calf compression/stockings		X	X		X					
Patient positioning			X, lower legs every 2–4 hours	X	X, lower legs every 2–4 hours, padding					
Hemorrhage management			X, TXA		X, TXA, consider TEG					
Vasopressor/Inotrope		X	X, noradrenaline		X, noradrenaline	X, noradrenaline				
ICU admission					X					
Fluid management	X	X, GDT- individualized	X, GDT	X, GDT	X, GDT	X, Maintenance rate 5-8ml/kg/hr, add 500 ml if needed			X, GDT	
Pain control/anesthesia		X, thoracic epidural, for 72 hr	X, Regional pain control, including epidural	X, Multimodal with neuroaxial, or TAP block	X, Regional pain control, including epidural	X, TIVA, no epidural			X, TIVA, no epidural or PCA	X, no epidural, consider nerve block or CWI
NGT		AVOID				AVOID				AVOID
Abdominal drain		AVOID, unless urinary resection				AVOID, unless urinary resection			AVOID	AVOID

ERAS indicates Enhanced Recovery After Surgery; Gyn Onc, gynecology oncology; TME, total mesorectal excision; hr, hour; OR, operating room; ICU, intensive care unit; NGT, nasogastric tube; PIV, peripheral IV; Hgb, hemoglobin; TXA, tranexamic acid; GDT, goal-directed therapy; TEG, thromboelestography; IV, intravenous; PCA, patient controlled anesthesia; CWI, continuous wound infiltration; TAP, transversus abdominis plane; TIVA, total intravenous anesthesia.

**Table 4:** Post-operative recommendations.

Post-operative	ERAS society		Collaborations		Expert opinions		Published studies			
Recommendation	Gyn Onc (30)	Urology – radical cystectomy (8)	PelvEx Guidelines (2)	Anesthesia Guidelines (43)	PelvEx Delphi (44)	TME protocol (45)	Harji et al (13)	Wan et al (47)	Huepenbecker et al. (12)	Nordkamp et al. (46)
Pain control		X, multimodal, epidural for 72hr	X, remove PCA by POD2, epidural by POD4		X	X, acetaminophen, and metamizole, prn fentanyl, continuous ketamine	X, remove PCA by POD2, epidural by POD4	X, fentanyl PCA thru POD1, then meloxicam, acetaminophen, prn oxycodone	X, Multimodal acetaminophen, ibuprofen, pregabalin, oxycodone, hydromorphone	X, Multimodal pain meds, CWI, metamizole, acetaminophen, avoid opioids
ICU transfer by POD2			X				X			
Nutrition	X, “early”	X, by 4 hr PO	X, by POD3			X, daily progressive goals for POD0-4, no limitations by POD4. Glucose checks thru POD2	X, by POD3		X, regular diet POD0, glucose control protocol	X, POD0 clears, progressing to solids by POD4.
Fluids								X, stop POD1	X, rate 40ml/h then stop after tolerating 500cc PO	
Foley catheter		X, remove by POD1 if no resection, unclear timing of stent and catheter if resection	X, remove by POD2 if no resection, POD7 if cystotomy, POD30 if cystectomy			X, remove with epidural, consider SPC if suspected prolonged retention	X, remove by POD2 if no resection, POD7 if cystomy, POD30 if cystectomy	X, remove by POD1	X, remove by POD1	X, Remove by end of procedure if no bladder surgery, SPC if anticipated prolonged retention
Abdominal drain removal			X, by POD8			X, by POD2 and <200ml serous per 24hr	X, by POD8			
VTE ppx	X	X, LMWH for 30 d	X, LMWH for 4 wks			X, enoxaparin for 28 d, compression stockings	X, LMWH for 30 d	X, enoxaparin POD1 thru discharge, and then risk based		X, for 30d
Mobilization	X, “early”	X, 2 h by POD0, 6 h by POD1	X, assisted standing and walking by POD1			X, daily progressive goal POD0-6	X, assisted standing and walking by POD1, goals by surgery type	X, by POD1	X, OOB on POD0, ambulation 8x/d POD1	X, progressing protocol to walking for POD0-3
PONV ppx						X, dexamethasone, granisetron, and prn metoclopramide				
Illeus prevention		X, chewing gum, Mg, multimodal				X, chewing gum, coffee, mag-ox, macrogrol		X, PEG and senna		X, chewing gum, laxatives
Daily weight						X				X
Discharge goals						X		X		
Transfusion									X, if Hgb <7g/dL	
Follow-up			X, offer psychological support			X, by POD30		X, by POD7		X, by POD30

ERAS indicates Enhanced Recovery After Surgery; Gyn Onc, gynecology oncology; TME, total mesorectal excision; ICU, intensive care unit; POD, postoperative day; VTE, venous thromboembolism; Ppx, prophylaxis; PONV, postoperative nausea and vomiting; PO, postoperative; LMWH, low molecular weight heparin; Mg, magnesium; PCA, patient control anesthesia; Hgb, hemoglobin; PEG, polyethylene glycol; OOB, out of bed; SPC, suprapubic catheter; CWI, continuous wound infiltration.

Today, most pelvic exenterations are performed via an open approach, with 3.6-13.1% performed minimally invasive (48, 49). Both colorectal and gynecologic oncology ERAS societies recommend minimally invasive approach when appropriate, as they are linked to the best surgical outcomes (28, 31, 42, 50). In a 2023 study in Germany, they performed a feasibility study using robotic surgery for completion of pelvic exenterations (14). They successfully performed a complete resection (R0) in all 13 patients in the study. A minimally invasive approach to PE could be a potential aspect of perioperative care to be explored in the future, in hopes of reducing complications of this very morbid procedure.

With total pelvic exenteration being very rare, and most institutions performing on average 1 case per year, this makes evaluation of surgical outcomes related to ERAS pathways very challenging (21). It will take a multi-institutional collaborative effort to properly address perioperative protocols. Some disputable questions remain regarding critical care admission, use of epidurals, patient controlled opioid pumps, abdominal drains, nasogastric tubes, bowel preparation, and oral VTE prophylaxis.

## Conclusion

Pelvic exenteration remains an extraordinarily complex and morbid procedure. We reviewed the limited data available in the literature about using ERAS protocols for these procedures. Even though highly complex and multifaceted, compliance to ERAS protocols has been demonstrated as feasible (12, 13, 46, 47). We, unfortunately, have not seen the benefits of standard ERAS pathways in predominantly gynecologic oncology pelvic exenteration. Further studies are required to provide best practices for minimizing complications, shortening length of stay, and maximizing patient outcomes.
